# Forced normalization and psychosis

**DOI:** 10.1192/j.eurpsy.2021.630

**Published:** 2021-08-13

**Authors:** M.T. Valadas, R. Mota Freitas

**Affiliations:** 1 Serviço De Psiquiatria, Unidade Local de Saúde do Baixo Alentejo, Beja, Portugal; 2 Departamento De Psiquiatria E Saúde Mental, Hospital do Espírito Santo de Évora, Évora, Portugal

**Keywords:** psychosis, Epilepsy

## Abstract

**Introduction:**

Epilepsy is associated with a wide range of psychiatric manifestations. Forced normalization occurs when the establishment of improved seizure control in a patient with previous uncontrolled epilepsy leads to the emergence of psychiatric symptoms, which include, among others, psychotic phenomena.

**Objectives:**

We aim to review the literature regarding the phenomenon of forced normalization and its association with psychosis.

**Methods:**

We performed an updated review in the PubMed database using the terms “forced normalization” and “psychosis”. The included articles were selected by title and abstract.

**Results:**

Psychosis is the most common behavioural disturbance in forced normalization, usually manifested as delusions and hallucinations. Forced normalization is more frequent in young female patients with drug‐resistant focal epilepsy. Antiepileptic drug treatment and epilepsy surgery are the most common triggers. Institution of antipsychotics and management of antiepileptic drugs are part of the treatment. Prognosis seems to be better in women, children and patients with generalized epilepsy, among other factors.

**Conclusions:**

Forced normalization is an overlooked entity, the pathophysiology of which remains largely uncertain. The recognition of forced normalization by psychiatrists is crucial for adequate patient treatment including pharmacological management and consultation with a neurologist

